# Effectiveness of the world anti-doping agency's e-learning programme for anti-doping education on knowledge of, explicit and implicit attitudes towards, and likelihood of doping among Chinese college athletes and non-athletes

**DOI:** 10.1186/s13011-022-00459-1

**Published:** 2022-04-26

**Authors:** Zhangyan Deng, Jinyang Guo, Dong Wang, Tao Huang, Zuosong Chen

**Affiliations:** 1 School of Education, Shanghai Jiao Tong University, Shanghai, China; 2 Department of Physical Education, Shanghai Jiao Tong University, Shanghai, China; 3 School of Kinesiology and Health, Capital University of Physical Education and Sports, Beijing, China

**Keywords:** Anti-doping education, WADA, Evaluation, Implicit attitude, fNIRS

## Abstract

**Background:**

This study aimed to evaluate the effects of the World Anti-Doping Agency's e-learning programme for anti-doping education on knowledge of, explicit and implicit attitudes towards, and likelihood of doping among Chinese college athletes and non-athletes.

**Method:**

Thirty-two young adults (including 16 college athletes) were recruited to receive the Athlete Learning Program about Health and Anti-Doping (ALPHA) intervention (Zh-hans version). Another 32 young adults were recruited for no-treatment control purposes. Before and immediately after the intervention, the ALPHA test, performance enhancement attitude scale, doping likelihood scale, and brief implicit association test (BIAT) were performed. Cortical activity during the BIAT test was monitored using a functional near-infrared spectroscopy instrument.

**Results:**

Significant intervention effects were observed for knowledge (*p* < 0.01, *η*^2^ = 0.21) and explicit attitude (*p* < 0.05, *η*^2^ = 0.12) but not for doping likelihood (*p* > 0.05; benefit situation: *η*^2^ = 0.04; cost situation: *η*^2^ = 0.02). Compared with the non-athletes, the college athletes reported lower doping likelihood scores in benefit situations (e.g., financial gain, *p* < 0.05, *η*^2^ = 0.10). Regarding the BIAT task, the experimental effect was successfully induced by different semantic associations between the concepts and the attitude (doping + like vs. doping + dislike). The mean reaction times (*p* < 0.01, *η*^2^ = 0.36) and error rate (*p* < 0.01, η^2^ = 0.34) in the doping-like block were higher than those in the doping-dislike block. Moreover, oxygenated haemoglobin (oxy-Hb) in response to BIAT interference in the temporoparietal junction-related channels was increased during the post-intervention test (*p* < 0.05, *η*^2^ varied from 0.09 to 0.16).

**Conclusions:**

The findings suggest that the online anti-doping education programme is partially effective among Chinese college athletes and non-athletes. Furthermore, our findings reflect enhanced cognitive control after the education intervention to suppress a prepotent implicit attitude towards doping.

**Supplementary Information:**

The online version contains supplementary material available at 10.1186/s13011-022-00459-1.

## Background

Due to the prevalence of doping and its negative consequences in sports, education strategies have received a great deal of attention for their role in doping prevention over the past decade [1, 2]. The China Anti-Doping Agency (CHINADA) recently introduced its local regulation rules and certified thousands of clean sport educators [3]. These initiatives outline a widespread offline education system that provides registered testing pools and international and national elite athletes with opportunities to obtain anti-doping education. Meanwhile, there is an urgent need for college athletes to obtain anti-doping education. First, doping is defined in the World Anti-Doping Code as the occurrence of one or more of the anti-doping rule violations. There are 11 anti-doping rule violations (ADRVs) that define a spectrum of behaviours, ranging from the presence of a prohibited substance to tampering doping control procedures. There are yearly reports of anti-doping rule violations among sub-elite athletes, including college athletes [4]. Second, some college athletes will follow elite athlete training routines, and others might become physical education teachers, coaches, or sport-related officers in the future. Currently, Chinese college athletes receive anti-doping education mainly from event-based e-learning courses on the China Anti-Doping Education Platform (CADEP). This platform mainly offers video-based online webinars. The content of these webinars focuses on anti-doping rules. The International Standard for Education advocates that anti-doping education should encompass awareness-raising (highlighting topics and issues related to clean sport), information provision (providing accurate, up-to-date content related to clean sport), values-based education (delivering activities focusing on developing personal values and principles, and ethical decision-making) and anti-doping education (anti-doping information building competencies in clean sport behaviours and informed decision-making). Compared with other components, the values-based education component has not received sufficient attention in the current anti-doping education system among Chinese sub-elite athletes. To fulfil the anti-doping education system in China, CHINADA cooperated with the World Anti-Doping Agency (WADA) in translating the 'Athlete Learning Program about Health and Anti-Doping (ALPHA)' seminar in 2020. ALPHA offers a pragmatic and interactive approach and represents a holistic educational aim alternative to video-based online webinars [5]. In addition to providing accurate knowledge about the 'World Anti-Doping Code', this seminar aims to form negative attitudes towards doping and offers practical help in high-risk scenarios [6]. It might further contribute to developing clean sport values and building informed decision-making among Chinese athletes.

With the development of outcome-based evaluations, researchers are paying increasing attention to the comprehensive effects of anti-doping education, including knowledge, attitudes, and behaviour [7]. The Knowledge, Attitude, Practices (KAP)/Knowledge, Attitude, Behaviour (KAB) model was first developed in the 1950s. This model was extensively used in health education and as a guide to understanding the mechanisms of health education for patient behavioural changes and patient health outcomes [8, 9]. Since the 1990s, this research framework has been introduced to the anti-doping education field. Indeed, researchers [10, 11] have posited positive relationships between attitudes and doping behaviour. In addition, health concerns were the components of the attitudes towards doping [12]. Knowing the health consequences of doping might contribute to forming a negative attitude towards doping [13]. Meanwhile, questions have been raised about the relationship between knowledge and behaviour. Although most anti-doping education programmes have had positive intervention effects on the knowledge of doping [14–18], the effectiveness of anti-doping education programmes on attitudes towards and the likelihood of doping remains controversial [14–16, 19–27]. However, few studies have simultaneously examined the potential effect of the ALPHA intervention on knowledge, attitudes, and behaviours [28]. Since this is the first time a values-based anti-doping education programme has been introduced to China, the effectiveness of this programme deserves to be clarified.

Existing studies of prohibited performance-enhancing substances have focused not only on athletes but also on non-athletes [29]. Taking health concerns and public perception into consideration, a wide range of anti-doping education initiatives have been developed. Several studies have assessed the effectiveness of these initiatives on knowledge of, attitudes towards, and likelihood of doping among non-athletes [14, 21, 26, 30–32]. However, there is no evidence on the implementation of anti-doping education interventions targeting Chinese non-athletes. As mentioned above, the content of CADEP is focused on anti-doping rules. This e-learning platform might not be suitable for non-athletes. Since WADA claims that ‘ALPHA represents a holistic, values-based approach and offers a pragmatic and positive alternative to the traditional approach to preventing doping’, the ALPHA intervention might effectively shape the knowledge of, attitudes towards and likelihood of doping among non-athletes. In addition, Codella and colleagues [30] recently launched a short-term anti-doping education programme ('Lotta al Doping') among Italian non-athlete samples and found that anti-doping education enhanced anti-doping knowledge after the intervention. The 'Lotta al Doping' and the ALPHA intervention share several domains of educational content, such as prohibited substances list, consequences of doping, forming negative attitudes towards doping, etc. Thus, the contents of ALPHA intervention could be understood by non-athletes.

Based on dual-process theories, human cognition can be described as a function of both an impulsive, automatic system (system 1) and a reflective, deliberative system (system 2). Explicit attitudes towards doping reflect outputs from the deliberative system [33]. However, implicit measurements are thought to tap into the impulsive system [34]. The brief implicit association test (BIAT) was recently modified to measure implicit attitudes towards doping [35–37]. Based on the semantic associations between concepts and attitudes, stimuli in the BIAT could be divided into congruent (e.g., flowers + like) and incongruent (e.g., insects + like) conditions. The semantic meaning of the concept interferes with attitude in the incongruent condition but not in the congruent condition. Participants have to suppress their prepotent response, which explains the longer reaction time in the incongruent condition [38, 39]. Thus, the BIAT could be postulated to control for social desirability. Meanwhile, questions have been raised about the usefulness of BIAT on whether this paradigm was adequately sensitive to measure implicit attitudes towards doping [40]. In addition, reaction time-based BIAT is an indirect measure that does not allow for conclusions as to whether behavioural performance reflects cognitive function related to doping. Neuroimaging techniques can be used to identify actual cognitive mechanisms underpinning doping attitudes. Indeed, using functional magnetic resonance imaging (fMRI), Luo and colleagues [41] found that a significant haemodynamic response was evoked in the incongruent condition of the implicit association test compared to the congruent condition. This suggested that the cerebral haemoglobin response was related to the implicit association test. Recent electroencephalogram (EEG) studies have suggested that several cortical areas, including the prefrontal cortex, are activated during the execution of doping BIAT tests [39]. Moreover, an additional experimental manipulation, such as faking, might enhance EEG activity in the prefrontal cortex and temporoparietal junction regions [42, 43]. However, EEG devices have poor spatial resolution [44]. Compared with EEG, functional near-infrared spectroscopy (fNIRS) is a promising neuroimaging technique that non-invasively measures changes in the concentration of oxygenated haemoglobin (oxy-Hb) and deoxygenated haemoglobin (deoxy-Hb). It analyses brain activity with a higher spatial resolution than EEG and is not restricted to scanning environments [45, 46]. An extensive literature of neuroscience studies shows that fNIRS can monitor real-time changes in the prefrontal cortex and temporoparietal junction regions [47, 48]. In addition, a recent review suggested that fNIRS is suitable for measuring brain activation changes in the framework of educational neuroscience [49]. To our knowledge, no fNIRS studies have examined the relationship between anti-doping education and implicit attitudes.

Given the research gap in the field, the present study had two main objectives. The first purpose was to evaluate the effect of the ALPHA intervention on knowledge of, explicit attitude towards, and likelihood of doping among Chinese samples. The second purpose was to clarify the effect of the ALPHA intervention on implicit doping attitudes, as measured by behavioural and neural activity during the BIAT test.

## Materials and methods

### Participants

A total of 64 participants (including 32 first-level athletes with at least 5 years of competition experience) were recruited from 3 universities in Shanghai, China (Table 1). All participants were recruited using a convenience sampling procedure and were balanced with the prior intention for group (intervention vs. control), age, gender, athletic event (team vs. individual sports), and competitive levels (athlete vs. non-athlete). None of the participants had taken the ALPHA seminar before. The study protocol was approved by the ethical committee at Shanghai Jiao Tong University (ethical code: H2020040I).

**Table 1:** Participants' characteristics

	**Intervention Group** ***N* = 32**		**Control Group** ***N* = 32**	
	Non-athlete	College athlete	Non-athlete	College athlete
Female %	50%	50%	50%	50%
Age	22.5 ± 2.94	20.7 ± 2.33	24.75 ± 3.64	21.81 ± 3.12
Competitive Level	Amateur	First-level athlete	Amateur	First-level athlete
Athletic Event		Team sports = 8		Team sports = 8
		Individual sports = 8		Individual sports = 8
**Note:** All first-level athletes previously participated in national youth games or tournaments				

The a priori sample size was calculated using G*Power 3.1 software (Universität Düsseldorf, Düsseldorf, Germany). A minimum of 36 participants were needed, given the type of experimental design (pre-intervention vs. post-intervention), a type I error of 5%, statistical power of 80%, a medium effect size of 0.3 [50, 51], and multiple groups (athlete vs. non-athlete; intervention vs. control) and response variables [7, 52].

### Experimental Design and Procedure

A quasi-experimental design (between-group variable: competitive level and group; within-group variable: time) was used in the present study. Before the baseline measurement, all participants obtained and signed an informed consent form.

During the baseline measurement, the participants were asked to complete a series of scales on their knowledge of, attitude towards, and likelihood of doping. Then, they completed the BIAT. These sessions took approximately 30 min.

The intervention group was invited to revisit the lab after two weeks, during which time they took the ALPHA seminar*.* After completing the ALPHA seminar, all participants were measured again using the same procedures as the baseline measurement. The control group received no anti-doping education intervention.

### Intervention

The Chinese version of the ALPHA was employed as an intervention programme developed by WADA (developer) and CHINADA (translator). The content of this seminar consisted of 8 modules: 1. the doping control process; 2. whereabouts of athletes; 3. therapeutic use exemptions (TUE); 4. results management; 5. medical reasons not to dope; 6. ethical reasons not to dope; 7. practical help to stay clean; and 8. how to deal with pressure. A previous study indicated that modules 1–4 provided accurate knowledge about anti-doping rules. Modules 5–6 explained why doping is a health risk factor and result in forming negative attitudes towards doping. Modules 7–8 adopted a novel delivery mode, including an 'if–then' plan, scenario-based learning, and video testimonials from elite athletes to offer practical help on how to stay clean and how to resist the pressure to dope [6, 28]. The ALPHA seminar was presented on a 24’’ computer screen at a viewing distance of approximately 80 cm. The delivery of this seminar was based on the default setting of the ALPHA seminar. All participants were asked to learn each module (from module 1 to module 8) in the same sequence, which took approximately 80 min. Screen recording software (EVcapture, Hunan, China) was used to monitor the learning process.

### Measures

To measure knowledge of doping, the Chinese version of the ALPHA test was used. This test was developed by WADA and consisted of 12 multiple choice questions (each with one correct answer). The content of this knowledge test was connected with the ALPHA seminar. The ALPHA scores were calculated by counting the number of correct answers [28]. As a caveat, it was difficult to calculate the *α* coefficient due to the multiple content dimensions of the ALPHA test. Taking content similarity and formal application into consideration [28], we carried out this survey despite these limitations to the research.

The Chinese version of the Performance Enhancement Attitude Scale (PEAS) assessed explicit attitudes towards doping. A back-translation method was used to translate the original 17 items into Chinese [53]. Consistency between the English and Chinese versions was evaluated by a group of researchers. Statements were rated on a 6-point Likert scale (1 = strongly disagree to 6 = strongly agree). Higher scores on the PEAS indicated a more positive attitude towards doping. The internal consistency of the PEAS was 0.91 [54], and the PEAS exhibited acceptable internal consistency in the pre-intervention (α = 0.80) and post-intervention tests (α = 0.90).

The doping likelihood scale was adapted from previous studies [55–57]. The participants were presented with a set of hypothetical doping scenarios and were asked to judge how likely they would use the doping in each scenario, which was rated on a 7-point scale (1 = not at all likely, 7 = very likely). The original scale has shown good internal consistency in benefit (*α* = 0.96) and cost (*α* = 0.97) situations [56]. A back-translation method was used to translate the English version of the doping likelihood scale into Chinese [53]. The Chinese version of the doping likelihood scale exhibited acceptable internal consistency in the pre-intervention test (benefit situation: *α* = 0.91; cost situation: *α* = 0.91) and post-intervention test (benefit situation: *α* = 0.92; cost situation: *α* = 0.88).

The picture-based doping BIAT was used for this study [35–37]. The BIAT task stimuli consisted of four types of pictures: doping (target category), healthy food (target category), like (attribute category), and dislike (attribute category). The participants were asked to judge the classification of the target categories and attribute categories. In the congruent block, 'doping' and 'dislike' shared a response key 'F' key on the keyboard, and 'healthy food' and 'like' shared the same response key 'J' on the keyboard. In the incongruent block, 'doping' and 'like' shared the same response key 'F' on the keyboard, and 'healthy food' and 'dislike' shared the same response key 'J' on the keyboard. The BIAT test begins with a training session in which the participants were taught to discriminate between focal doping and non-focal healthy food pictures. After this training session, the participants performed two congruent blocks (20 trials) and two incongruent blocks (20 trials) in the ABBA sequence. For each trial, stimuli were displayed in the centre of the screen until participants made the right decision, and the category labels were displayed at the top of the screen [39, 58]. For each block, an interval blank screen was displayed for 15,000 ms (ms). In a pilot study, 60 athletes were recruited to examine whether the general tendencies for the doping BIAT task could be reproduced in Chinese. The stimulus and procedure were identical to those in the current study. The participants could easily distinguish task categories (target: doping vs. healthy food; attribute: like vs. dislike) and make accurate responses in the BIAT task. The mean reaction time and error rate in the incongruent (doping-like) block were higher than those in the congruent (doping-dislike) block (reaction time: 937.43 ± 246.70 ms vs. 830.32 ± 226.04 ms, *F*(1, 59) = 14.04, *p* < 0.01, *η*^2^ = 0.19; error rate: 3.65 ± 4.67% vs. 1.99 ± 2.38%, *F*(1, 59) = 10.50, *p* < 0.01, *η*^2^ = 0.15).

### fNIRS Measures

**Fig. 1:**
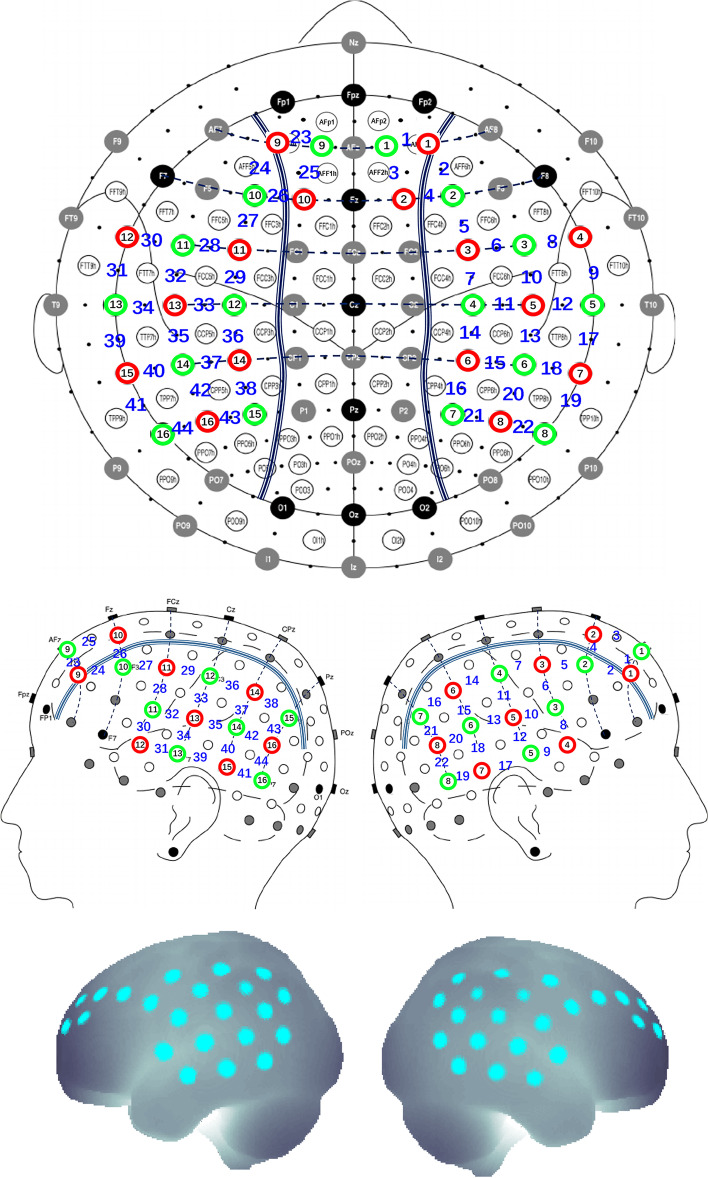
The layout of the optical source (red circles) and detector (green circles)

A multichannel fNIRS instrument (NIRSport 2, NIRx Medical Technologies) was used to monitor haemodynamic responses during the BIAT task (sampling rate = 4.45 Hz). Sixteen dual-wavelength sources (760 and 850 nm) and 16 optical detectors were placed on the 10/10 EEG positions (Fig. 1), which covered the frontal, parietal, and temporal regions (44 channels). The probe arrangement was finely adjusted to ensure that the distance between the source and the detector was 3 cm. The probabilistic estimation method was used to estimate the projection of each channel in the cerebral cortex [59, 60]. The spatial registration tools were obtained from Jichi Medical University.

The pre-processing procedure was identical to that in our previous study [61]. A bandpass filter (0.04–0.3 Hz) was used to remove baseline drift and physiological noise. Since deoxy-Hb signals have an inferior signal-to-noise ratio than oxy-Hb signals [62, 63], only the oxy-Hb signals were employed for further analysis. The mean values over the task period (5–45 s) were calculated for each channel and condition. In addition, the haemodynamic response was adjusted to the mean value of the baseline (from -2 to 0 s).

### Data Analyses

SPSS 20.0 (SPSS Inc.) was used to perform statistical analyses. Assumptions of data normality were examined graphically. The experimental effect of BIAT was first analysed by repeated-measures analysis of variance (ANOVA). This analysis was limited to the main effect of the task (incongruent vs. congruent) because the purpose of the ANOVA was to examine whether the general tendencies observed in the BIAT task could be reproduced. In addition, D-scores were calculated according to the standard scoring algorithm [35–37]. Then, we subtracted the oxy-Hb values collected during the congruent block from those collected during the incongruent block as interference effects. Finally, the scale scores, D-scores, and oxy-Hb interference data were assessed by a 2*2*2 repeated-measures ANOVA. Greenhouse–Geisser corrections were applied, where the assumption of sphericity was violated. The Bonferroni method correction was used to adjust multiple comparisons. Significance levels for all comparisons were set at *p* < 0.05 (two-sided). The effect size (η^2^) was calculated and categorized with 3 suggested cut-off points: small, less than 0.06; medium, 0.06 to 0.14; and large, greater than 0.14 [51].

## Results

To ensure consistency in the dependent variables, the participants in the control group were matched for athletic event and gender, with allocation at a 1:1 ratio (Table 1). In addition, an independent sample t test was used to compare pre-test scores between the intervention and control groups. No significant difference was observed between the intervention and control groups (all p > 0.05, Cohen's *d* varied from -0.02 to 0.15; Cohen suggested that *d* = 0.2 be considered a 'small' effect size [51]). Due to these consistencies in the dependent variables, all participants were pooled.

### Knowledge

A significant main effect for time (*F*(1, 60) = 14.49, *p* < 0.01, *η*^2^ = 0.20) and an interaction effect for group × time (*F*(1, 60) = 15.90, *p* < 0.01, *η*^2^ = 0.21) were observed on knowledge test scores. Post hoc tests showed that the observed interaction effect was driven by a significant increase in knowledge test scores in the intervention group (*p* < 0.01) compared with the control group. Knowledge test scores did not differ between athletes and non-athletes (*F*(1, 60) = 3.79, *p* > 0.05, *η*^2^ = 0.06). The descriptive results regarding the ALPHA test are shown in Table 2 and Supplementary Figs. 1 and 2.

**Table 2:** Descriptive results for the scale scores

	**Intervention Group**		**Control Group**	
	Non-athlete	College athlete	Non-athlete	College athlete
**Pre-intervention test**				
ALPHA Scores	9.50 ± 1.46	10.13 ± 1.31	9.06 ± 2.18	10.06 ± 1.12
PEAS Scores	30.50 ± 9.68	26.75 ± 7.50	29.43 ± 11.09	27.37 ± 8.48
Doping Likelihood Scores in Benefit Situation	2.74 ± 1.38	1.97 ± 0.98	2.72 ± 1.22	2.03 ± 0.92
Doping Likelihood Scores in Cost Situation	1.51 ± 1.02	1.25 ± 0.52	1.33 ± 0.43	1.31 ± 0.50
**Post-intervention test**				
ALPHA Scores	11.19 ± 0.75	11.13 ± 1.02	8.94 ± 2.02	10.12 ± 1.26
PEAS Scores	25.56 ± 12.48	23.00 ± 10.28	28.50 ± 11.46	27.75 ± 8.72
Doping Likelihood Scores in Benefit Situation	2.28 ± 1.19	1.61 ± 0.80	2.68 ± 1.30	2.10 ± 1.28
Doping Likelihood Scores in Cost Situation	1.34 ± 0.53	1.20 ± 0.50	1.28 ± 0.49	1.41 ± 0.65

### PEAS Scores

The results of the repeated-measures ANOVA pertaining to the PEAS scores indicated a significant main effect for time (*F*(1, 60) = 10.99, *p* < 0.01, *η*^2^ = 0.16) and an interaction effect for group × time (*F*(1, 60) = 8.48, *p* < 0.01, *η*^2^ = 0.12). Post hoc tests showed that the intervention group participants reported lower post-intervention test scores than pre-intervention test scores (*p* < 0.01). PEAS scores did not differ between athletes and non-athletes (*F*(1, 60) = 0.89, *p* > 0.05, *η*^2^ = 0.02). The descriptive results regarding the PEAS scale are shown in Table 2.

### Brief Implicit Association Test

**Fig. 2:**
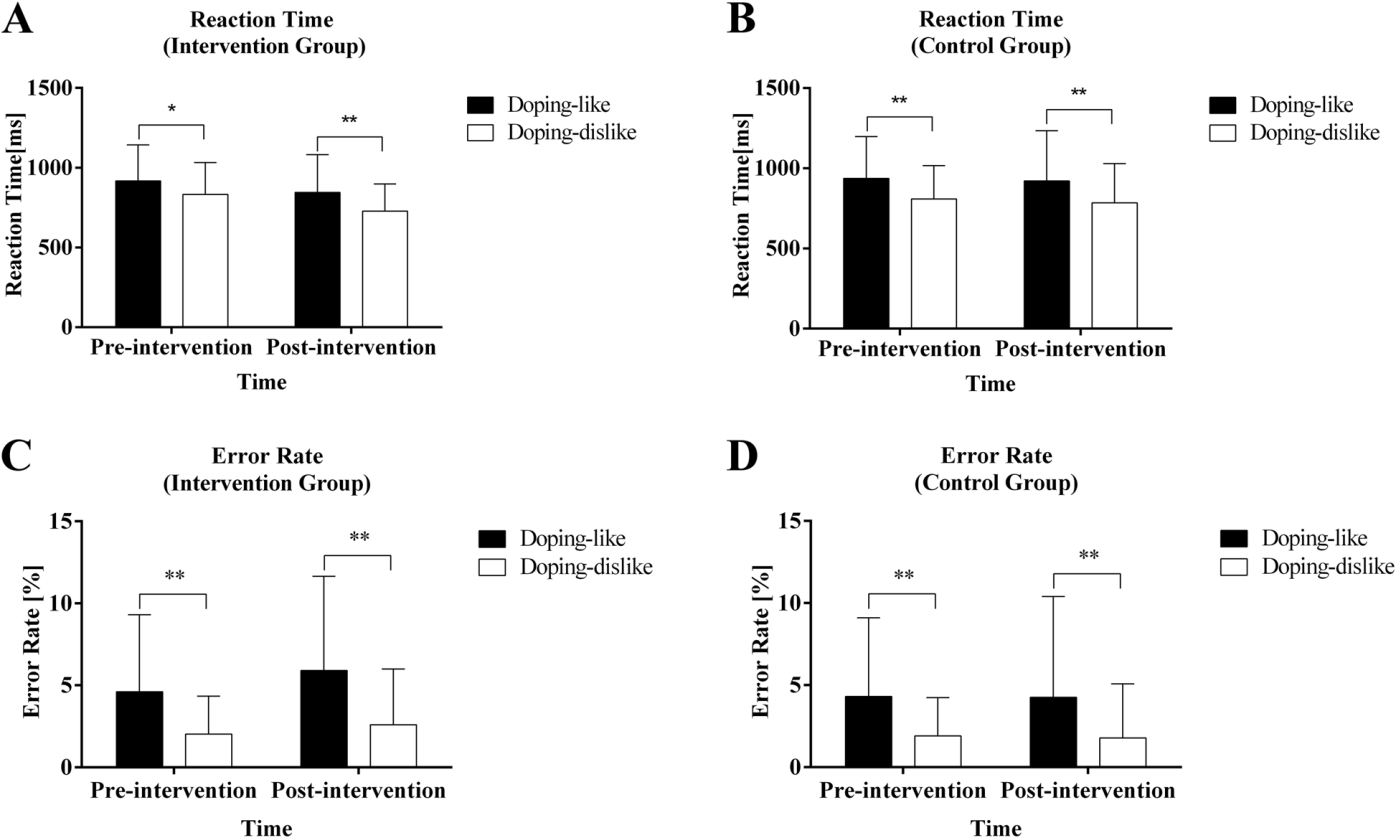
Behavioural Performance of BIAT Test. Comparison of reaction times (**A** and **B**) and error rates (**C** and **D**) of study participants between the incongruent (doping-like) and congruent (doping-dislike) conditions

**Fig. 3:**
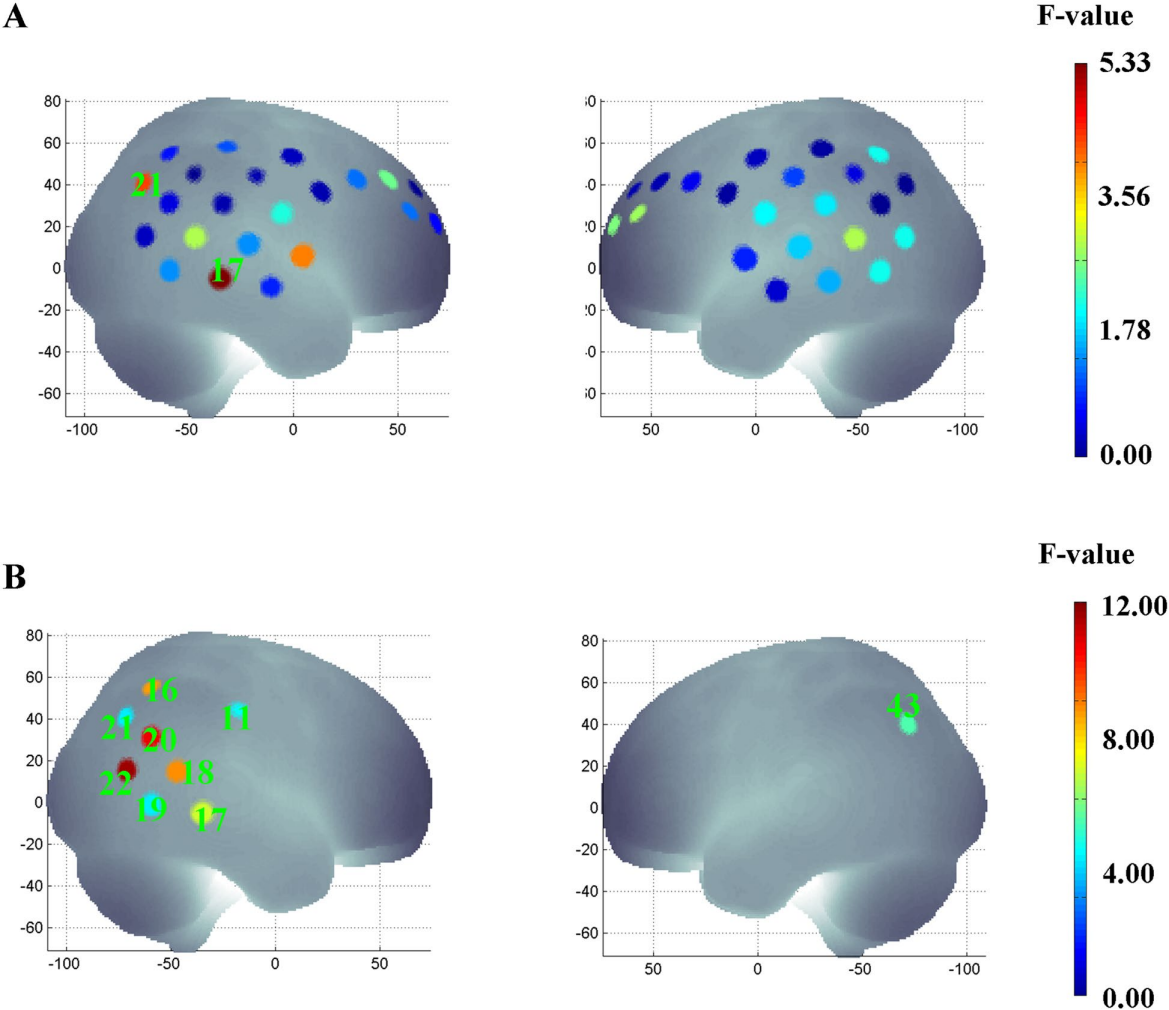
fNIRS result of BIAT test. F-map of oxygenated haemoglobin (oxy-Hb) concentrations between the incongruent (doping-like) and congruent (doping-dislike) condition (**A**), and F-map of oxy-Hb changes reflecting the interaction effect of Group × Time (**B**)

ANOVA for reaction time and error rate revealed a significant main effect of the experimental conditions. As shown in Fig. 2, the mean reaction time (*F*(1, 60) = 33.30, *p* < 0.01, *η*^2^ = 0.36) and error rate (*F*(1, 60) = 30.82, *p* < 0.01, *η*^2^ = 0.34) in the doping-like block were higher than those in the doping-dislike block. Meanwhile, a significant main effect of experimental conditions (doping-like > doping-dislike) was found in channels 17 (*F*(1, 60) = 5.33, *p* < 0.05, *η*^2^ = 0.12) and 21 (*F*(1, 60) = 4.31, *p* < 0.05, *η*^2^ = 0.09). These channels were approximately located at the temporoparietal junction (Fig. 3A).

Moreover, the participants' average doping attitudes as measured by their D-Scores in the BIAT displayed a negative effect towards doping. However, there was no significant intervention effect on the doping-related BIAT D-scores (-0.36 ± 0.50 vs. -0.42 ± 0.49, *F*(1, 60) = 0.66, *p* > 0.05, *η*^2^ = 0.01).

Significant group × time interaction effects were observed in the temporoparietal junction (channels 11, 16, 17, 18, 19, 20, 21, 22, and 43; all *p* < 0.05, *η*^2^ varied from 0.09 to 0.16) regions (Fig. 3B). Post hoc tests showed that haemodynamic responses in the temporoparietal junction regions were enhanced after the intervention (*p* < 0.05). In the control group, haemodynamic responses did not differ between the pre- and post-test.

### Doping Likelihood Scores

ANOVA results indicated a significant main effect of the competitive level (*F*(1, 60) = 6.99, *p* < 0.05, *η*^2^ = 0.10) in the benefit situation. The athletes reported lower doping likelihood scores in the benefit situation than the non-athletes. The descriptive results regarding the doping likelihood scale are shown in Table 2.

## Discussion

This study aimed to assess the effectiveness of the ALPHA seminar for Chinese college athletes and non-athletes. The results demonstrated that the education intervention induced significant changes in knowledge of doping and both explicit and implicit attitudes towards doping, but the likelihood of doping was not influenced by anti-doping education.

With regard to doping knowledge, our study revealed the acceptable accuracy (> 0.8) of the ALPHA test [28]. The ALPHA intervention enhanced knowledge about anti-doping rules and the consequences of doping. In line with Murofushi's [28] studies, athletes' responsibilities were not well understood by the participants. Supplementary Fig. 1 (Item 11) shows that all the participants who misanswered the question selected a unified option (option A). Several factors could explain this observation. First, option A was mentioned multiple times in module 4. In contrast, options B and C were barely directly mentioned in the same module. Thus, some participants might have chosen a familiar option (option A) rather than the correct option (option D: all of the above options). Second, given that the detection and deterrence system is only relevant to elite athletes, the responsibility for testing might have been ignored by college athletes and non-athletes. Regarding other items, the participants showed higher ALPHA scores and accuracy than those in a previous study [28]. Cognitive involvement might have contributed to these differences. Compared with a previous study [28], we controlled the learning process to ensure that the participants were involved in and completed all modules. This factor might have resulted in enhanced cognitive involvement during the learning process in the current study. Interactive constructive active passive (ICAP) theory suggests that a greater cognitive engagement level could promote the learning process [64]. Wadouh and colleagues [65] found that students in higher cognitive engagement classes acquired more knowledge than those in lower cognitive engagement classes. In addition, Förtsch and colleagues [66] found a positive correlation between cognitive involvement and knowledge acquisition.

Ntoumanis et al. [11] and Blank et al. [67] posited a positive relationship between attitudes and doping behaviour. Thus, the effectiveness of anti-doping education programmes on attitudes towards doping has been emphasised. With regard to explicit attituded, the ALPHA intervention reduced attitudes towards doping after the intervention. This finding was consistent with Barkoukis and colleagues [23, 31, 68], who found that education reduced attitude scores. Similarly, Lucidi and colleagues [30] found that education interventions induced stronger attitudes against doping. Unfortunately, the impact of anti-doping education on explicit attitudes towards doping remain controversial [7, 22]. Elbe and Brand [69] reported a reverse relationship between education and PEAS scores. Taken together, these findings raise the question of whether the intervention changed their attitudes or made participants feel bad about their attitudes and respond in a socially desirable way. It should be noted that these studies used different instruments (e.g., 17-item PEAS, 6-item PEAS, and the adolescent sport doping inventory) to evaluate the effectiveness of anti-doping education on doping attitudes. Since the content of these measures are different, the mixed performances might also be attributed to the measures used to assess attitudes. However, behavioural performance hardly identifies actual cognitive mechanisms underpinning doping attitudes. Thus, we used fNIRS equipment for the first time to examine the effectiveness of anti-doping education on implicit attitudes. With regard to implicit attitudes, reaction times and error rates in the congruent block (doping-dislike) of the BIAT task were lower than those in the incongruent block (doping-like), confirming that implicit attitudes were dissociated by these two task conditions. This finding is consistent with Brand and colleagues [35], who used a picture-based BIAT and found that the experimental setting evoked significant reaction time differences among athletes. The semantic meaning of the concept interferes with the attitude in the incongruent block but not in the congruent block, which may require cognitive resources to inhibit automatic or impulsive responses. When performing the BIAT task, this interference may result in greater activation of relevant brain areas in the incongruent block than in the congruent block. Indeed, the experimental setting resulted in greater activation of the temporoparietal junction area in the incongruent block (doping-like) than in the congruent block (doping-dislike) [41]. Moreover, the oxy-Hb response to BIAT interference in the temporoparietal junction was increased during the post-intervention test. These results indicated that the education intervention might have affected the neural mechanisms of cognitive control. Schindler and colleagues [42] suggested that enhanced activity in the temporoparietal junction during the BIAT may be responsible for suppressing an automatic response tendency towards doping. Since the temporoparietal junction is associated with cognitive control, we hypothesised that education might promote cognitive resource allocation to inhibit automatic response tendencies related to doping and result in socially desirable responses. This study presents the first experimental evidence on the neural substrates underlying the cognitive effects of anti-doping education.

With regard to doping likelihood, anti-doping education did not change the likelihood of doping scores in benefit or cost situations. On the one hand, the intervention effect of the ALPHA programme is partially acceptable. Since modules 7 and 8 offer practical strategies to resist group pressures, they probably decrease the likelihood of doping in such situations. Indeed, the mean score of Item 5 (doping likelihood scale: 'Encouraged by a coach or manager') was significantly decreased after the intervention. Meanwhile, we observed a noticeable but insignificant intervention effect in the benefit situation. On the other hand, several prominent predictors regarding doping behaviour were ignored by the ALPHA seminar. For example, it has been suggested that moral disengagement predicts the intention to use prohibited substances [70–73]. Recently, two anti-doping education intervention programmes that targeted moral disengagement were developed [19, 20]. These programmes significantly reduced doping likelihood among UK and Greek athletes. Thus, future anti-education programmes should be theoretically and evidence based.

Compared with the college athletes, the non-athletes reported higher doping likelihood scores in the benefit situation. Compared with UK college athletes [55], non-athletes had higher average doping likelihood scores in the same situation. One explanation for the group differences is that anti-doping values have emerged with the current athletic training culture. Thus, involvement in the athletic training routine might increase the sensitivity towards doping issues and diminish the likelihood of doping. A second explanation is the floor effect. In accordance with Ring and colleagues [55], participants reported low initial doping likelihood scores, making it almost impossible to further lower these values [11]. Except for doping likelihood, we could not observe any mediating effect of the group. When we considered the performance of non-athletes as a baseline condition, these results indicated that Chinese sub-elite athletes still lack knowledge. Therefore, it seems crucial to reflect the current anti-doping education system for Chinese sub-elite athletes.

As a holistic anti-education programme, the content of the ALPHA intervention approximately covers the components of awareness-raising, information provision, values-based education, and anti-doping education. Although, the ALPHA intervention enhanced knowledge about anti-doping rules and raised awareness of the doping issue, the current findings indicated that this e-learning programme was not effective in building clean sport value and preventing doping decisions. The findings of this study have some implications for doping prevention practices in China. First, it highlights the need to better tailor anti-doping education to Chinese cultural backgrounds. Although, CHNADA has launched large-scale anti-doping education activities [4], there has been very little published research on Chinese anti-doping initiatives and athlete values. It has been suggested that there are differences in the underpinning principles that guide athletes' behaviour. These principles might further mediate the effectiveness of values-based education. Accordingly, CHNADA needs to implement multifaceted prevention strategies based on Chinese characteristics. Second, future investigations are required to integrate other values-based components into anti-doping education programmes. The content of the anti-doping education programme could include moral cognition components, such as moral disengagement, to enhance the effectiveness of anti-doping education on doping likelihood. Third, traditional anti-doping education programmes tend to provide knowledge about anti-doping first rather than contributing to the formation of an attitude [6]. However, it has been suggested that a brief priming intervention on self-affirmation might enhance the effectiveness of anti-doping education [23, 68]. Since individual attitudes and beliefs mediate the effectiveness of education interventions [74], the anti-doping education programmes should include brief priming interventions to contribution to the formation of attitudes, beliefs, or perceptions in the initial session.

To our knowledge, this is the first study showing the relationship between anti-doping education and brain function in Chinese athletes. However, several limitations need to be acknowledged. First, the study lacked an active control group. Thus, we do not know how the intervention programme was effective. For example, the ALPHA programme was delivered via an interactive e-learning seminar. Given that CHINADA typically employs a variety of education activities (e.g., event-based education or in-person workshops), delivery modes and teaching strategies might modulate the effects of anti-doping education. These factors should be considered in future evaluations. Second, the PEAS is widely used to measure doping attitude. A recent systematic review opined that their finding ‘questions the validity of PEAS as a proxy for indexing doping behaviour’ [34]. Interestingly, improved internal consistency reliability of the PEAS scale was observed in the present study. These differences can be explained by the variance in scale scores. On the one hand, the variation in PEAS scores could be explained as an intervention effect. On the other hand, the statistical validity of the original scale could be questioned [75]. Indeed, Nicholls et al. [22] and Medina et al. [26] used alternative measures to assess doping attitudes. Future anti-doping intervention studies could include other explicit attitude measures. Third, the WADA launched a new anti-doping education and learning platform in January 2021. The contents regarding anti-doping rules, consequences of doping, and risks of supplements are greatly emphasised in the new seminars targeting talented/national/international athletes. Moreover, several elements, such as modules 7–8, which offer practical help, vocal commentary, and video testimonials from elite athletes, are removed. Taking these changes into consideration, the practical value of the current study regarding attitudes towards and likelihood of doping is restricted. Last, the study examined only the acute effects of anti-doping education. Therefore, future investigations are required to evaluate the long-term impact of the latest WADA anti-doping education programme.

## Conclusion

This study indicated that anti-doping education positively influenced knowledge of doping and both explicit and implicit attitudes towards doping. However, doping likelihood was not influenced by anti-doping education. This study represents the first attempt to elucidate the relationships between anti-doping education and brain function, which indicated that the education intervention promoted cognitive resource allocation to inhibit automatic response tendencies related to doping resulting in socially desirable responses rather than directly changing attitudes.

Taken together, the ALPHA intervention represents a valid preventive tool that successfully provides information and raises awareness about the doping issue. However, it was not effective in building clean sport value and preventing doping decisions among Chinese sub-elite athlete samples. Our findings suggest that there is an urgent need for robust evaluation of anti-doping education programmes. National anti-doping organisations should endeavour to tailor anti-doping interventions to particular cultural backgrounds. Multifaceted prevention strategies and values-based education programmes should be greatly emphasised in Chinese anti-doping initiatives that could protect athletes against doping initiation in the future.

## Supplementary Information

Additional file 1 **Fig. S1** The Alpha scale and the scores distribution of each question (intervention group)

Additional file 2 **Fig. S2** The Alpha scale and the scores distribution of each question (control group)

## Data Availability

The data and material are available upon reasonable request.

## Abbreviations

ALPHA: Athlete Learning Program about Health and Anti-Doping
BIAT: Brief implicit association test
CHINADA: China Anti-doping Agency
CADEP: China Anti-doping Education Platform
deoxy-Hb: deoxygenated haemoglobin
EEG: Electroencephalogram
FDR: A false discovery rate
fNIRS: Functional near-infrared spectroscopy
ICAP: Interactive constructive active passive
KAP: Knowledge, Attitude, Practices
KAB: Knowledge, Attitude, Behavior
oxy-Hb: Oxygenated haemoglobin
PEAS: Performance Enhancement Attitude Scale
ANOVA: Nalysis of variance
WADA: World Anti-Doping Agency
