# Supplementary material for: Effectiveness of the world anti-doping agency's e-learning programme for anti-doping education on knowledge of, explicit and implicit attitudes towards, and likelihood of doping among Chinese college athletes and non-athletes
Source: Subst Abuse Treat Prev Policy. 2022 Apr 26;17:31. doi: 10.1186/s13011-022-00459-1 (PMC9044811; doi:10.1186/s13011-022-00459-1)
Supplement: Supplementary file 1 — Additional file 1 Fig. S1 The Alpha scale and the scores distribution of each question (intervention group) [file 13011_2022_459_MOESM1_ESM.docx]

## Supplementary







**Supplementary Figure 1.** The Alpha scale and the scores distribution of each question (intervention group)
